# Electrophoresis-Enhanced Detection of Deoxyribonucleic Acids on a Membrane-Based Lateral Flow Strip Using Avian Influenza H5 Genetic Sequence as the Model

**DOI:** 10.3390/s140304399

**Published:** 2014-03-05

**Authors:** Jui-Chuang Wu, Chih-Hung Chen, Ja-Wei Fu, Huan-Ching Yang

**Affiliations:** Department of Chemical Engineering, Chung Yuan Christian University, Chung Li, Tao Yuan 32023, Taiwan; E-Mails: troy1987515@hotmail.com (C.-H.C.); prewave@hotmail.com (J.-W.F.); dm89kimo@yahoo.com.tw (H.-C.Y.)

**Keywords:** membrane-based lateral-flow detection, DNA detection, electrophoresis, avian influenza, biosensors

## Abstract

This study reports a simple strategy to detect a deoxyribonucleic acid (DNA) on a membrane-based lateral flow (MBLF) strip without tedious gel preparation, gel electrophoresis, and EtBr-staining processes. The method also enhances the detection signal of the genetic sample. A direct electric field was applied over two ends of the MBLF strips to induce an electrophoresis of DNAs through the strips. The signal enhancement was demonstrated by the detection of the H5 subtype of avian influenza virus (H5 AIV). This approach showed an excellent selectivity of H5 AIV from other two control species, *Arabidopsis thaliana* and human PSMA5. It also showed an effective signal repeatability and sensitivity over a series of analyte concentrations. Its detection limit could be enhanced, from 40 ng to 0.1 ng by applying 12 V. The nano-gold particles for the color development were labeled on the capture antibody, and UV-VIS and TEM were used to check if the labeling was successful. This detection strategy could be further developed to apply on the detection of drug-allergic genes at clinics or detection of infectious substances at incident sites by a simple manipulation with an aid of a mini-PCR machine and auxiliary kits.

## 1. Introduction

Detection of a DNA molecule can be performed by a costly but precise automated DNA sequencer [1], complicated and heavily instrument-dependent sequence-mining microarray biochips [2], or the Polymerase-Chain Reaction (PCR) [3]. The PCR process is so far known as the standard and basic procedure for efficiently and rapidly amplifying the signal of a genetic sequence by duplicating its specific DNA segment in a cost-effective manipulation. However, the processes after PCR, such as gel preparation, gel electrophoresis, EtBr-staining, and UV exposure are generally considered as both tedious and time-consuming steps. In addition, the PCR is also blind to the mutation occurring within the internal sequence. In regarding to these considerations, the rapid membrane-based lateral-flow (MBLF) detection is the best candidate to accelerate the post-PCR operations, particularly for the demands of detecting drug-allergy genes in the clinic or infectious substances at incident sites in a short-time.

MBLF detection is one of the most important tools used for rapid medical diagnoses and public-health research activities. It has been a popular platform for rapid tests since its first introduction in the late 1980s for pregnancy tests. MBLF detection offers low cost and operational simplicity for end users. It also allows untrained personnel to operate in an environment where access to laboratory instrumentation is limited or unavailable [4–6]. Such advantages quickly brought the MBLF technology to market by a small investment and it was applied in medical clinics in a short time frame. The application of MBLF detection to H5N1 analytes in samples taken directly from patients is a good example. Other competitive technologies currently being developed, for instance, array-based biochips or biosensors, still cannot replace this valuable technology.

Many researchers have reported various applications of MBLF using immunoassay for the detections of staphylococcal enterotoxin B [7] and ricin [8] to ensure food safety, botulinum neurotoxin for the study of the exocytosis mechanism of cells [9,10], viruses O, A and Asia 1 for serotype-specific foot-and-mouth diseases in animal husbandry [11,12], and deoxynivalenol [13] for the control of contamination in agricultural products, and sulfonamides to detect drug abuse in animals used for food [14]. Current MBLF technology has been combined with multiplex-nested PCR to detect various antibodies [15,16], antigens [17], and allergens [18,19].

MBLF has also been used to detect genetic signatures for acquiring essential information at the nucleic-acid level by rapid assay [20]. The deoxyribonucleic acids of *Papilloma* virus [21], *Legionella* and *L. pneumophila* [22], and Taura syndrome virus [23] all have been effectively detected using the MBLF technology. The most manifest advantage of this detection approach over direct detection on the original infectious substances is that researchers are entirely free from the risk of handling pathogens during the in-lab R&D processes. However, the big drawback of the above reports was that they all adopted complicated molecular techniques to enhance their detection signals.

**Figure 1.**
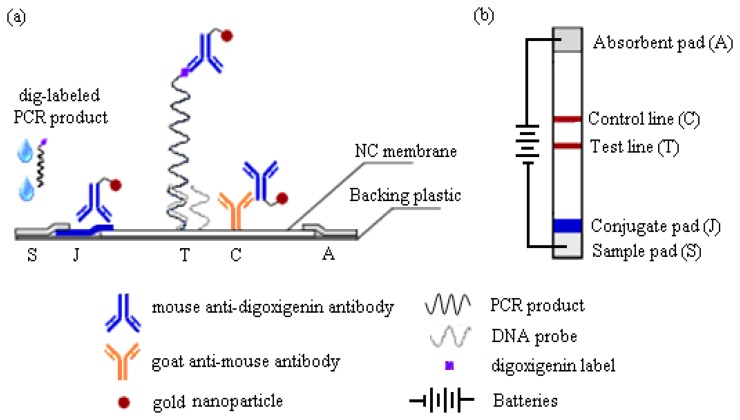
Assembly and assay of an electric-field enhanced MBLF strip. (**a**) Side view and (**b**) top view of the assembly. Gold tagged mouse anti-digoxigenin antibody loaded on the conjugate pad served as the reporter antibody. Rabbit anti-mouse IgG antibody served as the control antibody.

This work reports a detection strategy that not only eliminates all post-PCR steps, but confirms the DNA sequence and simultaneously enhances its detection signals on the MBLF strips using an electric field. The MBLF strips immobilized with the H5 AIV model oligonucleotide probe were used to demonstrate how to effectively recognize the desired genetic sequence by unaided eyes, unlike real-time PCR, which is generally understood as a rapid detection approach without gel electrophoresis, but still needs an aid from instrument, e.g., UV/VIS or fluorescence label, for read out. As shown in Figure 1a, the MBLF detection assembly was composed of five elements: (1) sample pad (S) to be loaded with DNA analyte and later uniformly released onto the neighboring conjugate pad; (2) conjugate pad (J) to be loaded with the report antibody to interact with the DNA analyte upcoming from the sample pad; (3) absorbent pad (A) to wick fluid and keep flow continuous along the strip; (4) a porous nitrocellulose (NC) membrane to induce lateral flow and to immobilize with the capture antibody; and (5) backing plastic card to adhere all above elements together. The oligonucleotide probe for H5 AIV was printed on top of the test line (T), which was printed in advance with 1X streptavidin. The rabbit anti-mouse IgG antibody was printed at the control line (C) as the capturing antibody to capture the report antibody as a validation check of the tests. As shown in Figure 1b, the top view of Figure 1a, a necessary number of 1.5 V batteries was connected in series to generate a desired direct electric field to induce the DNA analyte to flow through the capture zone. The detection assay and how this voltage connection enhances a DNA detection signal will be described later in more detail.

## 2. Experimental Section

### 2.1. Materials

The genetic sequence of H5 AIV from H5N1 was adopted as the study model. It also worked as the PCR template for the analyte preparation. Including the primers for PCR cycles and oligonucleotide probe immobilized on the membrane, all DNA sequences were synthesized by Purigo Biotech (Taipei, Taiwan). For brevity “H5 AIV” will refer the H5 sequence from the subtype H5N1 in the remainder of the paper. Like the H7N9 AIV recently spreading over mainland China, H5N1 is a highly infectious pathogen, generally spreading among poultry and birds. The outbreak in 2004 and the later reappearance of H5N1 caused human infections, deaths and anxiety. Several efforts have been made to detect this fatal pathogen [24] and cultivate effective vaccines to counter it [25].

*Arabidopsis thaliana* plasmid was adopted as the first species source of DNA negative-control target. It was the first plant genome to be sequenced and has become a popular model organism in plant biology and genetics [26,27]. The genetic sequence of this plant was cloned and notated as pda13015 (252b) in this study. The second species for the negative-control target is a human gene. Its PCR product was cloned and notated as PSMA5 (432b). The human PSMA5 is also a popular genetic model. A recent report indicated that it exists mainly as tetramer [28]. These two genetic targets are suitable as the negative models, since they are two very different species from the avian influenza and their genetic sequences have already been well studied. These two negative genetic targets were also synthesized by Purigo Biotech. The corresponding information of all genetic targets can be found in Table 1. The sequences of the DNA probe and the corresponding PCR primers of the genetic targets are listed in Table 2.

Rabbit anti-mouse IgG printed as the control line was obtained from USBiological (Salem, MA, USA). After being labeled with gold nanoparticles, mouse anti-digoxigenin antibody played the role of the report antibody and was purchased from Novus (Littleton, CO, USA). The gold nanoparticles, with an average size of 32 nm, were provided by Taiwan Advanced Nanotech (TaoYuan, Taiwan).

The essential materials for PCR are detailed as follows: Taq polymerase was purchased from NEB (Herts, UK) and dNTP from Amersham (Pittsburgh, PA, USA). Ethidium bromide for staining agarose gel, and TBE buffer, for running the gel, both were obtained from BioBasic (Markham, Ontario, Canada). The PCR products were purified by using a QIAquick purification kit (Qiagen, Valencia, CA, USA).

Nitrocellulose membrane, 5 μm AE98, used to load the oligonucleotide probes as the detection surface, was purchased from S&S (Boston, MA, USA). As other three-dimensional bio-compatible materials, e.g., an aerogel we recently developed [29,30], nitrocellulose provides a large internal surface area to adsorb much more biological analytes [31] than the traditional 2D surface of biosensors. The sample pad, #33 glass, and conjugate pads, #16S, were also purchased from S&S. The absorbent pads, #CF6, were obtained from Whatman (Pittsburgh, PA, USA). Plastic backing cards were purchased from Adhesives Research (Glen Rock, PA, USA). Sodium chloride, used for making SSC buffer, sucrose, for modifying the conjugate pads, zinc chloride, Triton-X, bovine serum albumin, magnesium and Tris base for making the antibody storage buffer, were all purchased from BioBasic.

### 2.2. Instruments

A TLC dispenser, Model Linomat 5 (CAMAG, Muttenz, Switzerland), was used to spread oligonucleotide probe and antibodies onto the membrane. The final membrane-pads assembly was cut into strips by the cutter, Model JS-101 (Jih Shuenn Electrical Machine, Taichun, Taiwan). The PCR machine, Model PC-320 (Astec, Tokyo, Japan), duplicated genetic samples for detection. The signal bands of the agarose gel were read on GeneFlash (Syngene, Frederick, MD, USA). UV/VIS (Jasco, V-530, Tokyo Japan) and Transmission Electron Microscopy (TEM, Jeol 2010) were used to differentiate between the antibodies before and after they were labeled with the gold nano-particles. The concentrations of genetic analytes were measured by a NanoDrop1000 spectrophotometer (Thermo Fisher Scientific, Hudson, NH USA). A low temperature centrifuge (Hettich 16R, Tokyo, Japan) was used to separate the gold-labeled antibodies from buffer. A desiccators (D-60C, Moisture Buster, Taichun, Taiwan), was used to store the nitrocellulose membrane. A HP4800 Scanner scanned the detection signals from the membrane. Commercially available 1.5 V commercial batteries were connected in series to generate direct electrical fields. The quantitative analysis of the signal intensities was carried out by genepixPro^®^ software.

### 2.3. Procedure

#### 2.3.1. Preparation of Running Buffers and Report Antibody

In a standard procedure, 20X SSC was prepared by dissolving 175.3 g of sodium chloride and 88.2 g of sodium citrate in ddH_2_O. The pH value of the solution was adjusted to 7.6 using HCl/NaOH. The final solution was prepared by diluting the original to 1L using ddH_2_O.

The report antibody, 0.2 mL of 50 μg/mL mouse anti-digoxigenin antibody, was mixed with the gold solution to label gold nanoparticles. After storage at 4 °C for 2 h, the solution was centrifuged at 4 °C and 14,000 rpm for 10 min. After disposal of top clear solvent, the gold-labeled antibody was added to 0.2 mL of 0.01 M Tris buffer as a wash. Another centrifugation and disposal cycle was then performed to obtain the final gold-labeled antibody, which was then saved in the lateral-flow buffer at 4 °C. The performance of the gold labeling was evaluated by UV/VIS.

#### 2.3.2. Immobilization of Oligonucleotide Probe and Antibody on Membrane

The NC membrane was first affixed onto the plastic backing card. Streptavidin was dissolved in sucrose to a concentration of 1.0 mg/mL and then loaded into a 100 L syringe. The syringe was loaded onto the TLC dispenser to print a 5 cm-long line in 20 L of the streptavidin solution. At the research stage, this study utilizes the TLC dispensing to provide a cost-effective operation prior to the final commercial production of test strips, which normally needs at least 1,000 L per run. The 20-mer biotinlayted DNA probe was dissolved in 2× SSC to a concentration of 400 mM, and then 4 L were dispensed on top of the previous streptavidin line to generate the test line. By the same procedure, 1 cm apart from the test line, the control line was printed with 1 mg/mL of rabbit anti-mouse IgG. The finish-printed NC membrane was then stored in a desiccator maintained at RH% of 30%–40%, for at least one day.

Prior to assembling NC membrane with the other necessary pads, the conjugate pad was immersed in 5% sucrose for 5 min and dried at room temperature for 1 day. All elements were then assembled in the following order onto the plastic card: NC membrane, conjugate pads, absorbent pads, and sample pads, as the relative positions shown in Figure 1. The finished assembly was finally cut into 5.9-cm long and 5-mm wide strips.

It is improper to immobilize a molecular marker, e.g., the marker ladder of gel electrophoresis, because of the limited space on the strips. The marker ladder will not be meaningful as the test line is already fixed at a certain position.

#### 2.3.3. Preparation of DNA Samples and Electrophoresis of Agarose Gel

To prepare the single-stranded (*ss*) DNA analytes, a standard asymmetric PCR was conducted in the procedure described as follows: an unequal proportion of primers were first mixed with PCR necessary substances. The forward primer, in a concentration of 10 μM and volume of 2 μL, and the reverse primer of 0.2 μM and 2 μL were mixed with 1 μL of 50 ng/ μL template, 5 L of 10X PCR buffer, 2.5 μL of 5 mM dNTP, and 36.5 μL ddH_2_O. After addition of 1 μL of 1.25U/μL Taq, the mixture was quickly loaded into the PCR machine. Each PCR cycle was set as denaturization 95 °C for 5.5 min, annealing 50 °C for 1 min, and extension 72 °C for 1 min. In total 40 cycles were conducted and the final product was purified using the purification kit.

Standard agarose gel in 2% of concentration was prepared by dissolving 0.6 g of agarose powder in 30 mL of 0.5X TBE. The solution was microwaved and then cast in an electrophoresis tray for 20 min. The DNA samples were mixed in 1:1 with an electrophoretic blue dye and loaded into the gel grooves. 100 V was applied for 30 min to allow electrophoresis. The gel slabs were then stained with ethidium bromide and images were acquired by exposing the gel to a UV light.

#### 2.3.4. The Detection Assay and Image Acquisition

As shown in Figure 1, the standard detection assay used a sandwich configuration with the target DNA captured between a ligand-specific primary antibody and a sequence-specific oligonucleotide probe. Mouse anti-digoxigenin antibody tagged with 32-nm gold nanoparticles served as the detection reporter and was loaded in advance on the conjugate pad. As the digoxigenin-labeled H5 AIV PCR product flowed through the conjugate pad, they would undergo a protein affinity binding of the digtoxigenin ligand with the mouse antibody to form a complex molecule. The resultant complex molecule then kept flowing toward the 20-mer DNA probe immobilized on the test line. The DNA probe was designed to be sequence complementary with the *ss*DNA (358b) H5 AIV target, which was amplified by the PCR product as mentioned earlier. The DNA probe should have no signal appeared when any negative-control genetic target was loaded. To validate all tests, rabbit anti-mouse IgG was dispensed as the control line to capture the report mouse antibody. Commercially available AA-size 1.5 V batteries were used as the direct electricity source.

In a typical run, the H5 AIV genetic analyte was first diluted to the desired concentration using the running buffer. Twenty-five μL of the analyte was added onto the sample pad and subsequently flowed onto its neighboring conjugate pad. On the conjugate pad, the analyte conjugated with the mouse anti-digoxigenin antibody to form the H5 AIV-antibody complex. For the scenarios of other two negative-control genetic analytes, *i.e.*, *Arabidopsis thaliana* and human PSMA5, there was no complex molecule formed, so that the anti-digoxigenin antibody will move alone to the control line. For the detection of H5 AIV genetic analyte, the H5 AIV-antibody complex migrated onto the NC membrane and was subject a capillary flow in the interstitial space of the membrane. Continuing along this flow path, the complex was captured by the oligonucleotide probe on the test line because of sequential complementation. An absorbent pad placed at the distal end wicked fluid away from the membrane to keep the flow continuous. The detection signals were finally scanned by the HP scanner and saved as images. The images were later retrieved to measure the signal intensity by the commercial software genepixPro^®^. The commercial scanner was found the best way to save qualified images for signal-intensity analysis in this study.

To keep a direct electric circuit passing through the membrane, another 25 μL of running buffer was simultaneously dropped at the position near to the absorbent pad when the analyte was loaded onto the sample pad. The running buffer would then flow toward two sides, one toward the absorbent pad and the other toward the test line. As the buffer stream encountered the analyte stream somewhere around the test line, a continuous liquid phase was form along the MBLF strip to conduct the direct electricity provided by the batteries.

## 3. Results and Discussion

### 3.1. Analysis of Gold-Nanoparticle Labeling

**Figure 2.**
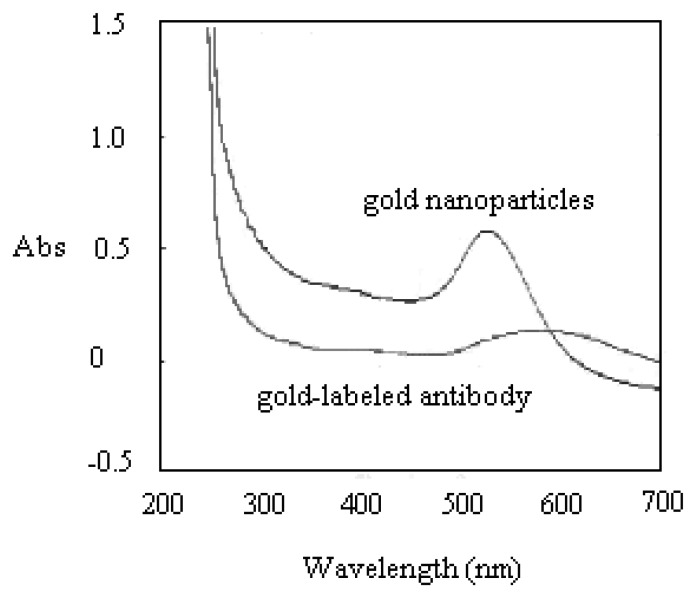
UV/VIS analysis of the gold-labeled antibody. The peak became flattened and the peak wavelength shifted from 524 nm to 560 nm, after the mouse anti-digoxigenin antibody was labeled with the nano-gold particles.

**Figure 3.**
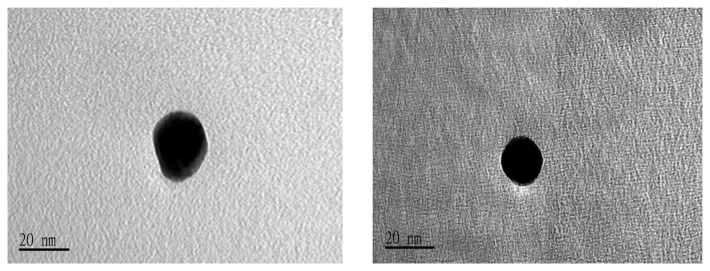
TEM analysis of the gold-labeled antibody. Left: a gold nanoparticle after being labeled onto antibody molecules with observable shadow around the gold sphere; Right: a gold nanoparticle before being labeled onto antibody molecules.

Prior to running the detection on the H5 AIV PCR products, the antibody reporter was verified the presence of its gold tag by UV/VIS. The UV/VIS measurement identified the gold-labeled antibody from its unlabeled format. This investigation was mainly undergone by checking the variation of spectrum shape and shift of the peak position. If gold nanoparticles are associated with other larger molecules, the wavelength of the absorbance peak will shift toward the Infrared (IR) side. As shown in Figure 2, the UV/VIS spectrum showed a flattened peak, shifting from wavelength 524 nm to 560 nm after the gold nanoparticles were successfully tagged onto the antibody reporter. Figure 3 shows the TEM analysis of the gold-labeled antibody. A 3∼4 nm-thick shadow ring could be observed around the gold sphere particle after it was labeled onto antibody molecules, with the comparison of the condition of no labeling.

After the verification of antibody reporter, the rapid detection on the genetic sequence of the as-prepared PCR products was then conducted by running the MBLF tests.

### 3.2. The Selectivity Test

**Figure 4.**
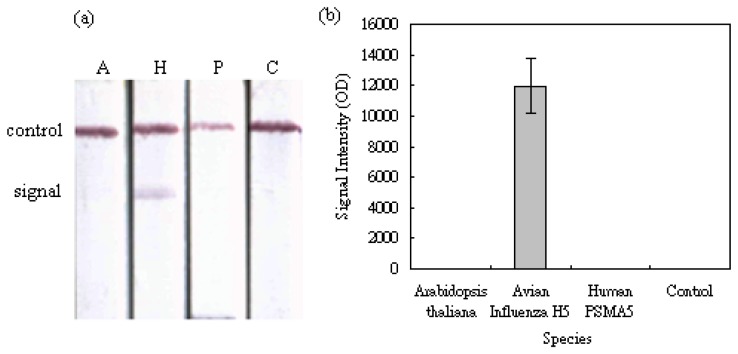
The selectivity test of the MBLF Strips. (**a**) MBLF test on the genetic sequences of (A) *Arabidopsis thaliana*, (H) H5 AIV, and (P) human PSMA5 PCR products, respectively, from left to right. Each analyte was loaded with 200 ng. The control strip (C) on the farther right was loaded with the running buffer only. A 12 V electric field was applied and the strip width was 5 mm. (**b**) Quantitative analysis of the signal readings.

When evaluating a functioning biosensor, the capability of selecting its specified biological target from other non-specific ones is always the first criterion and the most important indication of the sensing performance. The selectivity of our MBLF strips for identifying the H5 AIV analytes from *Arabidopsis thaliana* and human PSMA5 was carried out by detecting 200 ng of each DNA analyte on a 12 V electric field. As shown in Figure 4a, a signal was obtained from the H5 AIV genetic analyte while a clean background was observed for the two negative-control species, indicating our MBLF strips have an excellent selectivity to identify the H5 AIV genetic sample from other species. Figure 4b shows the quantitative analysis of the signal intensity of this investigation. After being subtracted from the membrane background, the signal intensity of the H5 AIV genetic sample in the chart showed a good identification from other two species. The control strip (C), running in plain buffer, validated this experiment.

**Figure 5.**
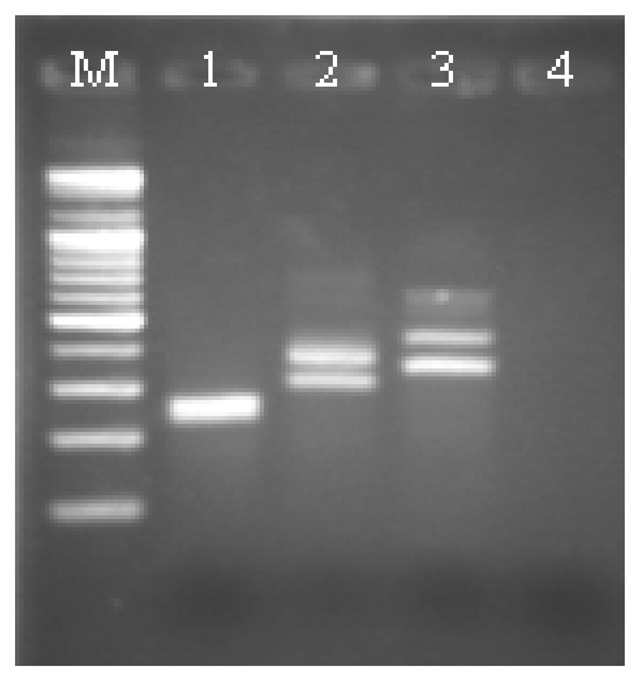
The corresponding gel electrophoresis of the PCR analytes for the selectivity tests. Channels M: the ladder DNA marker; 1: *Arabidopsis thaliana* (252 b); 2: H5 AIV (358 b) 3: PSMA5 (432 b); and 4: negative control. Double bands on each channel indicated the presence of both single and double stranded DNA analytes in the PCR products. *Arabidopsis thaliana*'s on Channel 1 obviously was too short in size to display discrete bands on this gel.

The corresponding electrophoresis gel of this test was shown in Figure 5. Double bands appeared in each gel channel representing that the asymmetric PCR products were successfully produced. Because of its coiled configuration, a single-stranded DNA normally migrates slower than its double-stranded counterpart. This characteristic caused the gel to show two separated bands for the DNA mixtures. However, this sort of discrete bands will be merged when the length of DNA analytes was short, as shown in Channel 1 for the *Arabidopsis thaliana* analyte.

Because it will be an enormous and costly task to verify the cross-reactions from all subtypes of avian influenza, a separated research effort from this study would be more general and meaningful to investigate how the signal intensities are affected by the sequence-mutation positions and mutation base numbers.

### 3.3. Signal Enhancement on MBLF Strips by an Electric Field

**Figure 6.**
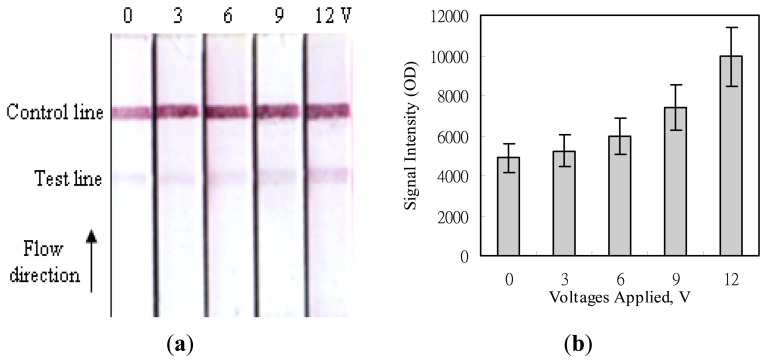
Intensity enhancement of detection signals on the H5 AIV genetic sequence by an electric field. (**a**) Image of the strip test. Each strip was applied various voltages, from 0 to 12 V. The strip width was 5 mm and the quantity of the H5 AIV analyte loaded was 200 ng. (**b**) Quantitative analysis of the signal intensities.

Figure 6 shows the images acquired after applying various voltages to the MBLF strips for the detection of the H5-AIV genetic sequence. The experiment was conducted by adding an identical amount of 200 ng H5-AIV PCR product onto all strips but applying different voltages of 0, 3, 6, 9, or 12 V over the membrane strips. The signal intensities on the test lines were found much lower than those on the control lines. That was because the detection mechanism on the test lines were based on the strand-to-strand DNA hybridization, which was with a weaker interaction force than the ligand-receptor affinity on the control lines. But the readers still can easily tell that the higher the voltage was applied, the more signal intensity was enhanced. This simple strategy of voltage application not only easily enhances the signal intensity of the MBLF tests, but also it eliminates the tedious procedures of gel electrophoresis, the post-electrophoresis ethidium-bromide staining, and UV exposure for verifying the DNA analytes and as well avoids consumptions of time and materials. In addition, the genetic sequence can be directly recognized by the immobilized DNA probe. This recognition function of a DNA sequence is so far absent in the traditional PCR procedure, which only confirms the sequence on two ends of the desired DNA segment by annealing two sequence-matched primers. It will ignore a new sequence if a mutation occurs within the desired segment. Since this study mainly intended to demonstrate the strategy for signal enhancement by a voltage application, we did not further investigate how many sequence bases of mutation that can be recognized by the MBLF tests.

**Figure 7.**
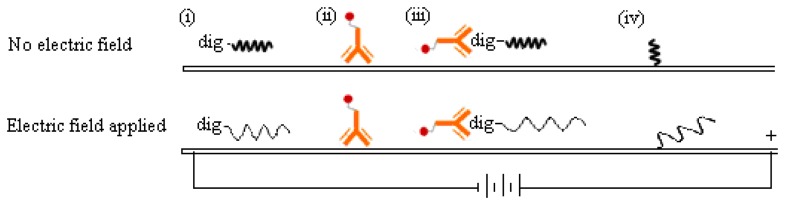
Schematic of configuration variation of single-stranded DNA's on the electric-induced MBLF strips: (top) no electric field; (bottom) DNA molecules are stretched by a direct electric field; molecules (i) dig-tagged *ss*DNA analyte; (ii) gold nanoparticle-labeled antibody reporter; (iii) the complex molecule of the previous two substances; (iv) immobilized *ss*DNA probe for recognizing the analyte.

The reason why a direct voltage enhanced the DNA detection signal on MBLF strips is unclear, but we guessed it could be attributed to the change of the analyte-probe interaction mechanism at the test line. As illustrated in Figure 7, the negative charges carried on the DNA analyte were attracted by the cathode located ahead when it migrated on the membrane strip. Meanwhile, the spatial configuration of the DNA analyte could vary by this electrical attraction. The DNA analyte would be stretched from the original coiled configuration to a linear one as it approached its DNA probe. Since the DNA probe was immobilized on the membrane, it could also be stretched into a linear configuration by the electric force. Consequently, this analyte-probe pair could have more contact area at the test line than that without voltage applied. This hypothesis would be reliable because the *ss*DNA probe was immobilized at one end. The other end would have a great chance to be stretched, but still stayed at the same immobilized spot. The protein-DNA complex, *i.e.*, the digoxigenin-labeled *ss*DNA analyte bound with the anti-digoxigenin antibody, could also be stretched into a linear format. As flowing through the membrane and simultaneously exposed to an electric force, this complex's shape was basically analogous to a chain (the *ss*DNA) dragging a shot (the antibody). The shot was much heavier than the chain in molecular weight and unlike to carry charges in the pH-neutral running buffer. Since the orientation of this chain-shot configuration was under controlled by the electric field, the antibody thus was dragged behind the DNA analyte. The antibody would therefore have a less probability, than the no-electric-field scenario, to interfere in the complementary hybridization undergone between the analyte and probe DNA.

### 3.4. The Sensitivity and Repeatability Tests of Electric-Field Enhanced MBLF Strips

**Figure 8.**
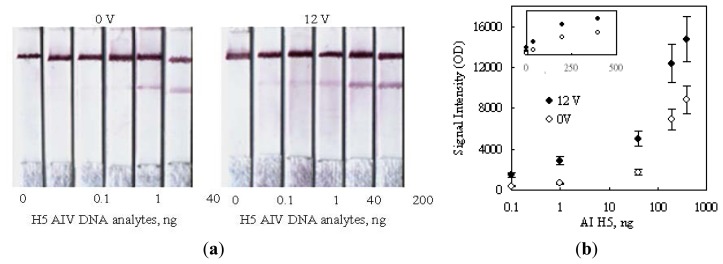
The sensitivity test of electric-field enhanced MBLF strips. (**a**) Left: detection sensitivity on MBLF strips without voltage applied; Right: 12 V was applied. Width of all strips was 5 mm. (**b**) The corresponding quantitative analysis of the signal intensities. The top small inset was identical to the big one but plotted on a linear scale for the horizontal axis. The big chart was plotted in a logarithm scale to show the detailed information of the low-quantity analyte.

Figure 8a shows the strip images obtained after performing a series of detections on various amounts of H5 AIV target. The color developed under a no-voltage-applied condition was obviously not detectable in the presence of 40 ng (339 femtomole for 358b) H5 AIV analyte. Since the 0.1 ng sample also had no signal, there is no reason to challenge a further lower dilution. The side-by-side comparison of the color developed by 12 V indicated that a detection limit of 0.1 ng (0.85 femtomole, 358b) DNA was achieved, which was lower than the 50 femtomole reported in the literature using the technology of deoxynucleotide-labeled gold nanoparticle [32] and 1 ng using the up-converting phosphor technology (UPT) for the detection of PCR products of human papilloma virus type 16 (HPV16) [21].

As shown in Figure 8b, the signal intensities on strips were also measured in a unit of optical density (OD) and plotted against the amount of H5 AIV analyte loaded. The result was plotted in a logarithm scale for the horizontal axis, in order to display the detail information for low analyte concentrations. As expected, the signal intensity correspondingly increased as the analyte concentration increased, and the 12 V data obviously showed an enhanced signal intensity as compared with the 0 V ones. However, the information for higher analyte concentrations became unclear using this display. This drawback could be corrected by re-plotted the result in a regular linear scale as the top small encompassed chart in Figure 8b. This replot interestingly did not show linearity. The data for two voltages individually trended toward a saturated reading as the analyte concentration increased. This signal-saturation trend implied that the binding sites on the test line were finite and their capturing ability became less and less efficient as the dose of the analyte increased, no matter whether a voltage was applied or not. The readers may check the slopes of the tangent lines along the imagined curve fitting the data points for each voltage. The slopes became more and more flattened along the increasing direction of the analyte dose. This dose response could be attributed to the steric effect created by hybridized DNA's on the neighboring unhybridized binding sites.

As reported in the literature, the steric effect created by a hybridized DNA could interfere with other approaching unhybridized ones to access their binding sites [33,34]. In this study, the hybridized complexes of H5-AIV DNA could also hinder their neighboring unhybridized DNA probes to contact with the upcoming complexes. Consequently, the unhybridized DNA probes on the test line had less and less chances to hybridize with the approaching DNA analyte as more and more spaces were occupied by the captured analyte.

Secondly, we also paid attention to the upward shifting of the saturation trend as 12 V was applied. This shift should be mainly due to the enhancement of the hybridization efficiency caused by the configuration re-orientation of DNA probes in an electric field, as previously mentioned, but the steric effect might also be diminished as the captured DNA analyte was re-orientated in parallel with the flow direction to yield its side spaces.

**Figure 9.**
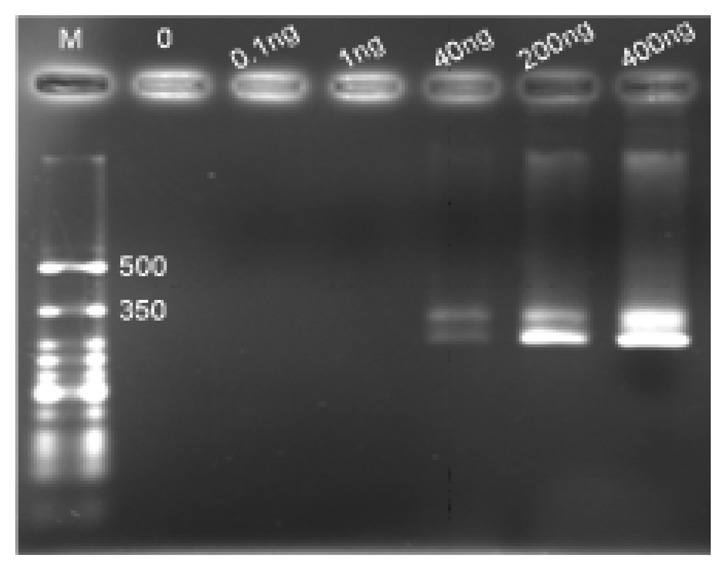
The agarose electrophoresis of the H5 AIV analytes for the sensitivity test. Channels M: *ss*DNA marker. Referring to the marker, the top band of each channel indicated that H5 AIV *ss*DNA was present, whereas the bottom band indicated *ds*DNA. The signals can be clearly recognized down to 40 ng.

As shown in Figure 9, the corresponding gel electrophoresis was performed to provide a reference of the detection limit. The same PCR products as those running on the MBLF strips on this gel were only detectable at 40 ng, indicating that the voltage-applied MBLF strips provided more sensitive detection performance than the traditional gel electrophoresis, which adopts the insertion of ethidium bromide into the grooves of DNA strands as its sensing reading appearance. The MBLF strip guaranteed itself a good substitution for the gel electrophoresis.

**Figure 10.**
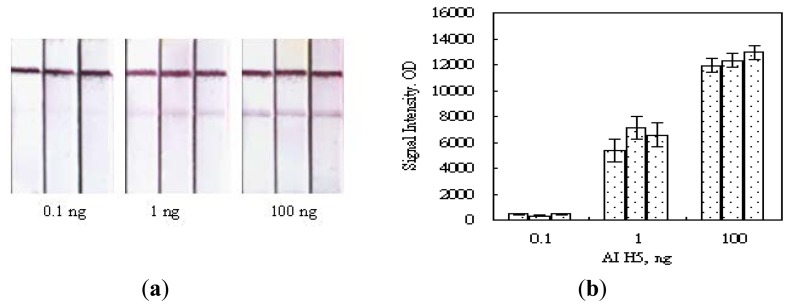
The repeatability of the MBLF strips with a voltage applied. Three replicate strips were use in the experiment for three different doses of H5 AIV analyte, 0.1, 1, and 100 ng. 12 V current was applied to all strips.

The signal repeatability of electric-field enhanced MBLF strips was also investigated by selecting three H5 AIV concentrations to run on three replicate strips for each concentration. As shown in Figure 10, the H5 AIV analyte was loaded in 0.1, 1, and 100 ng onto MBLF strips and the detection signals were analyzed. A consistent result was received for these three sets of replicates with an acceptable strip-to-strip repeatability of 4∼15% of CV%, indicating this method offers reliable performance.

### 3.5. The Joule Effect on the MBLF Strips

**Figure 11.**
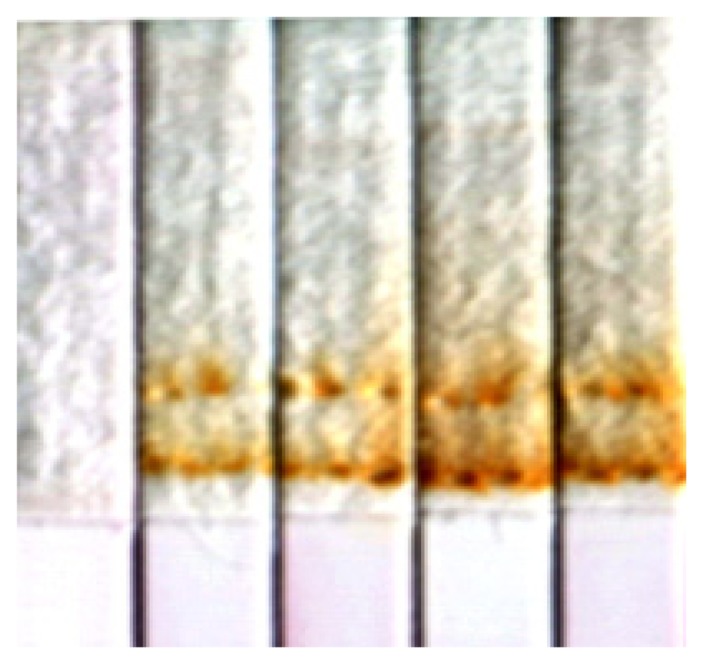
Typically observed burned-brown absorbent pads in this study due to the Joule effect. The leftmost strip has no voltage applied and the remaining strips received 12 V. The burned area can be seen around the clamping teeth. The strip width was 5 mm.

The signal intensity of the rapid MBLF detection in this study was observed to be enhanced by a direct electrical field. However, electric voltages higher than 12V were not further pursued because the Joule effect was observed from the burned-brown absorbent pads at 12 V, as shown in Figure 11. The Joule effect frequently occurring in gel electrophoresis has been known to cause a temperature rise and an uneven distribution across the gels, such that the gel matrix is deformed and the interpretation of the signals is consequently confusing. The impact of the Joule effect on the MBLF strips is still unclear, but neither deformation of membrane, test lines, control lines or the strip assembly nor shifting of test or control lines were observed in this study. Nonetheless, the most practical and advice is given here is to keep the electric voltage under 12 V to prevent the detection operation from a potential fire hazard. The other reason to utilize 12 V as the standard voltage for enhancing the MBLF detection is because this size of battery is already commercial available. A general mini 12 V battery tablet equipped in a compact set will good enough to provide the required electricity for the disposable MBLF assembly.

## 4. Conclusions

The most important outcome of this work is the development a DNA detection strategy on MBLF strips that skips all post-PCR steps and the discovery that the sensitivity of MBLF tests can be enhanced by simply applying a voltage over the membrane strips. To the best knowledge of the authors, this is the first report of such a technique using a MBLF device to enhance the detection of nucleic acids. MBLF has a great potential to be further developed into a commercial device for detecting drug-allergy genes at clinics or infectious substances at incident sites by a simple manipulation with an aid of a mini-PCR machine and auxiliary kits. In addition, this detection approach can be further developed to incorporate an in-parallel screening assay for a mixture of genetic samples by using a multiple DNA-probe array on the MBLF strips. The MBLF device will be capable of simultaneously and rapidly identifying various pathogens' genetic signatures on the same membrane panel.

## Figures and Tables

**Table 1.** The DNA analytes and their gene information.

**Genetic Target**	**Length (Bases)**	**Definition (Gene Bank)**
pda13015	252	Arabidopsis thaliana clone RAFL15-32-O04 (R20961) putative protein kinase (At1g76370) mRNA, partial cds. Riken Database BT003995.
PSMA5	432	Human Proteasome (prosome, macropain) subunit, alpha type, 5; NCBI Database gi 2311094, ref NM_002790.2
H5 AIV	358	Avian Influenza, type H5N1, Gene Bank S68489, A/turkey/England/50-92/91. It is only for the H5 from the H5N1 found from turkey in England in 1991.

**Table 2.** Probe and PCR primers used in this study and their sequences.

**Sequence**	
H5 AIV	It is only for the H5 from the H5N1 found from turkey in England in 1991.
forward primer	5'-digoxigenin -GCCACTCCACAATATACACCC-3'
**reverse** primer	5'-CAAATTCTCTATCCTCCTTTCCAA-3'
probe	5'-biotin-CAAATTCTCTATCCTCCTTT-3'
*Arabidopsis thaliana*	
forward primer	5'-digoxigenin-CTATAACCGAGATGTGCCTAAA-3'
**reverse** primer	5'- GTACAAACATTAATTATGCGG-3'
**Human** PSMA5	
forward primer	5'-digoxigenin-TGATAAAGCCAGAGTGGAGACA-3'
**reverse** primer	5' -TTCTTCCTTTGTGAACATGTGG-3'
